# Supplementary figures and images for: VapC toxins promote the pathogenesis of Rickettsia heilongjiangensis by cleaving essential RNAs from both Rickettsia and its host
Source: PLoS Pathog. 2025 Jul 30;21(7):e1013380. doi: 10.1371/journal.ppat.1013380 (PMC12321070; doi:10.1371/journal.ppat.1013380)

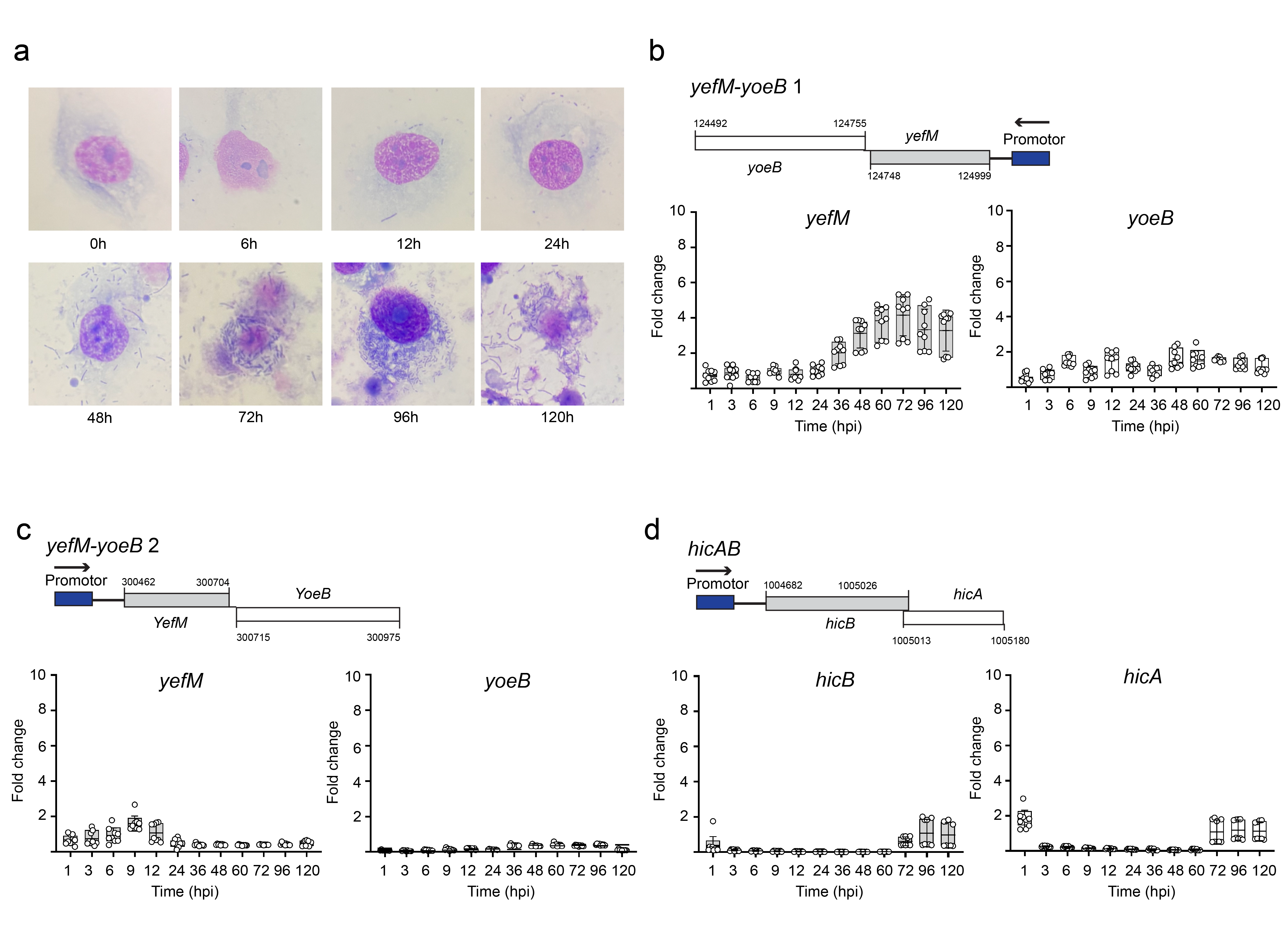

Supplement: S1 Fig — (a) Diff quick staining to show the rickettsial multiplication. (b-d) Genomic localization of yefM-yoeB and hicAB TA modules and their transcription level during bacterial growth. Gene transcription levels were measured after normalization to levels of ompB. The data was shown as the fold change in comparison to 0 hpi. Data with the mean ± SD are from n = 3 independent experiments, each with three technical replicates. (TIF) [file ppat.1013380.s001.tif]

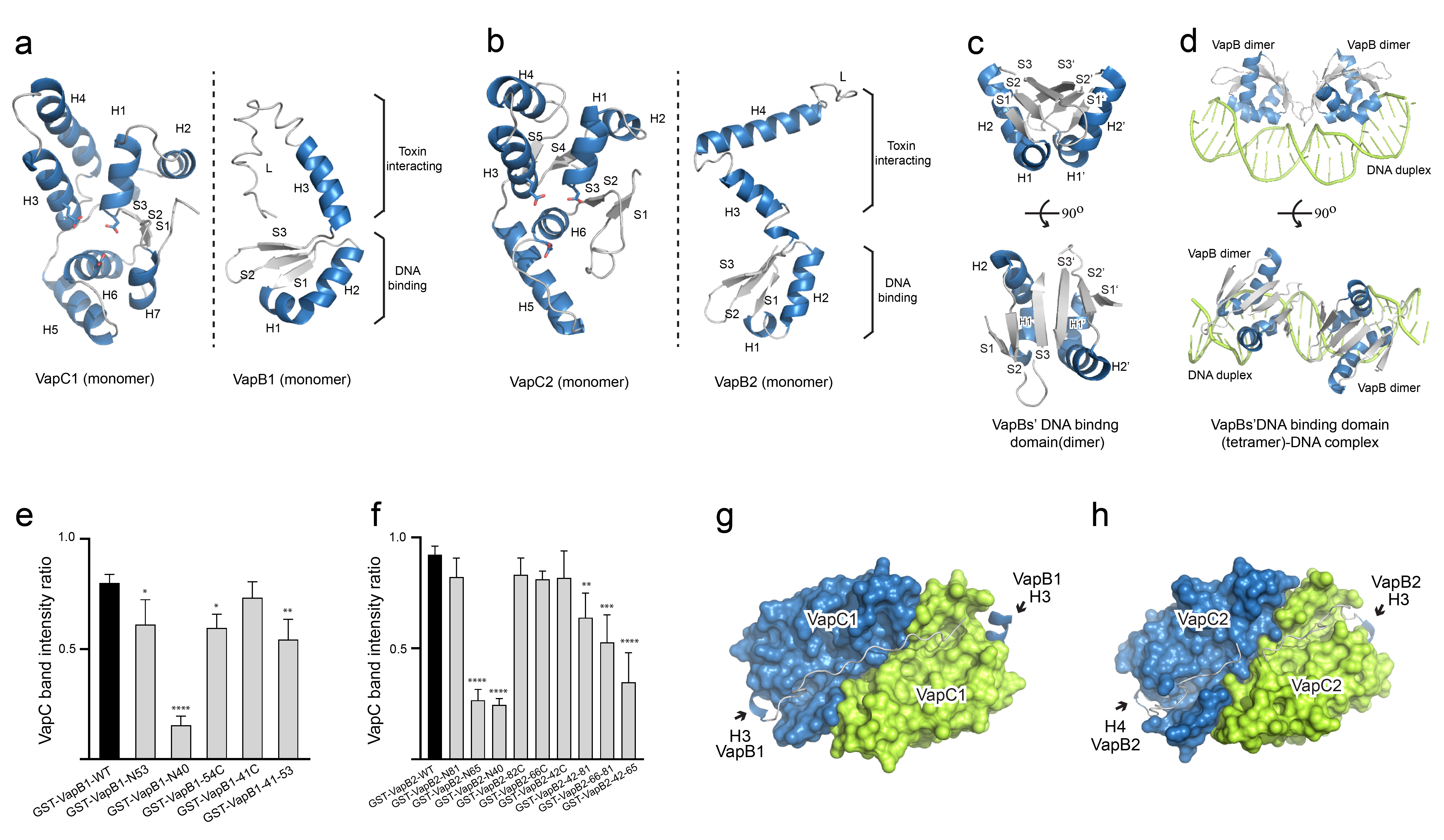

Supplement: S2 Fig — Protein structures were calculated in SWISS-MODEL based on Alphafold DB models. All output models had average model confidence (pLDDT) scores greater than 0.95. (a) and (b) Cartoon diagrams of the VapB and VapC monomers. Secondary structure elements were labeled, and colored in blue (α helices) and grey (β strands), respectively. The residues in the active sites were shown as sticks. The C-terminal toxin interaction domain and N-terminal DNA-binding domain were indicated. (c) The dimeric phd-like DNA binding domain of two VapB antitoxins. (d) Two orthogonal views of DNA-binding model of VapB tetramer. (e) Relative densitometric ratios of VapC1 bands normalized to the control (lane 9, Fig 2d). (f) Relative densitometric ratios of VapC2 bands normalized to the control (lane 14, Fig 2e). Data in (e) and (f) represent mean ± SD from three independent experiments. Statistical significance (*p < 0.1, **p < 0.01, ***p < 0.001, ****p < 0.0001) was determined by unpaired t-test comparing to wild-type. (g) and (h) Top views of the VapB C-terminus interacting with the VapC dimer. The VapC dimer was shown as a surface model, and the VapB C-terminal region was shown as a cartoon model. The C-terminal loop from VapC1 as shown in (e) was long enough to interact with two VapCs simultaneously. (TIF) [file ppat.1013380.s002.tif]

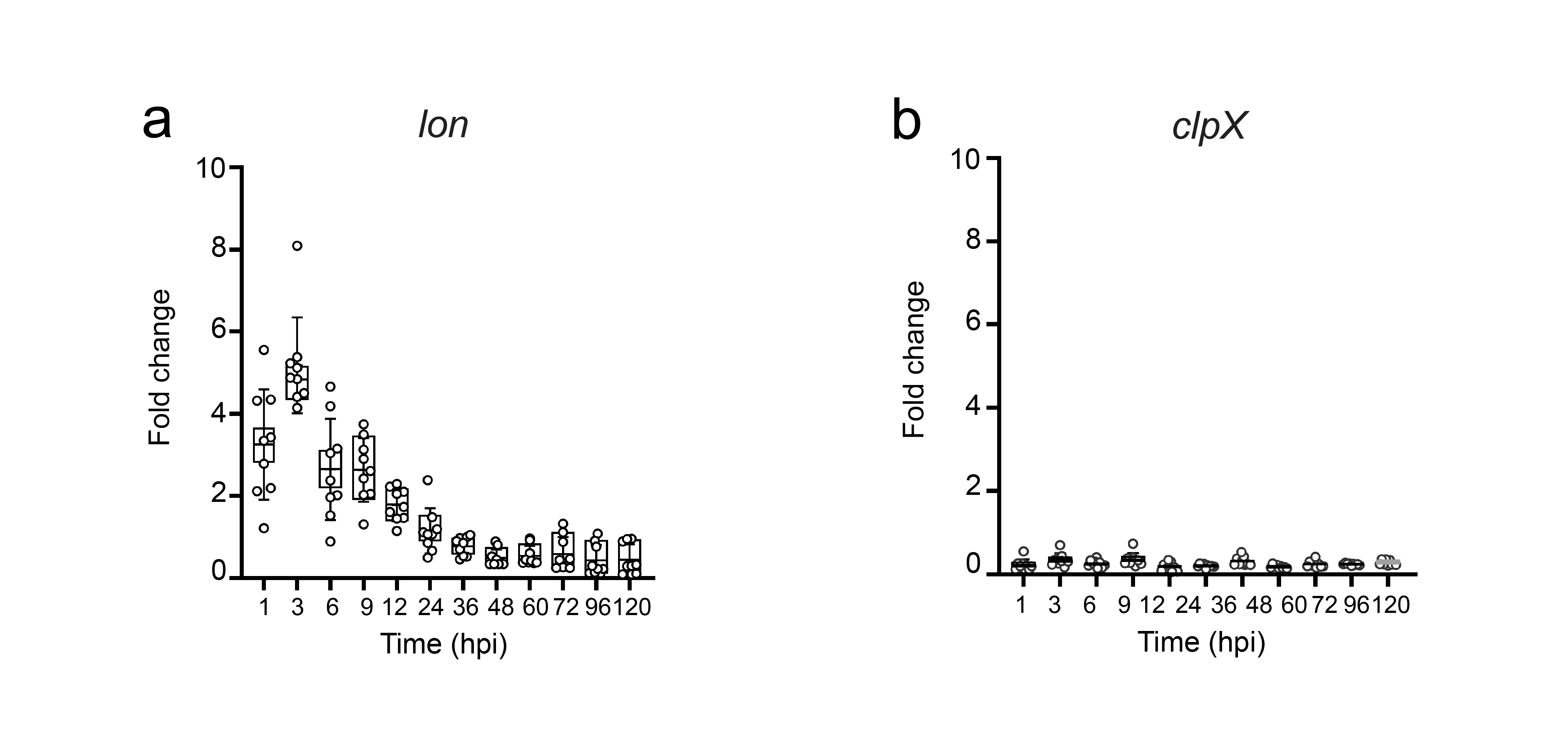

Supplement: S3 Fig — (a, b) Transcript levels of Lon and ClpX proteases, normalized to ompB expression, presented as fold change relative to 0 hpi. Data represent mean ± SD from three independent experiments, each performed with three technical replicates. (TIF) [file ppat.1013380.s003.tif]

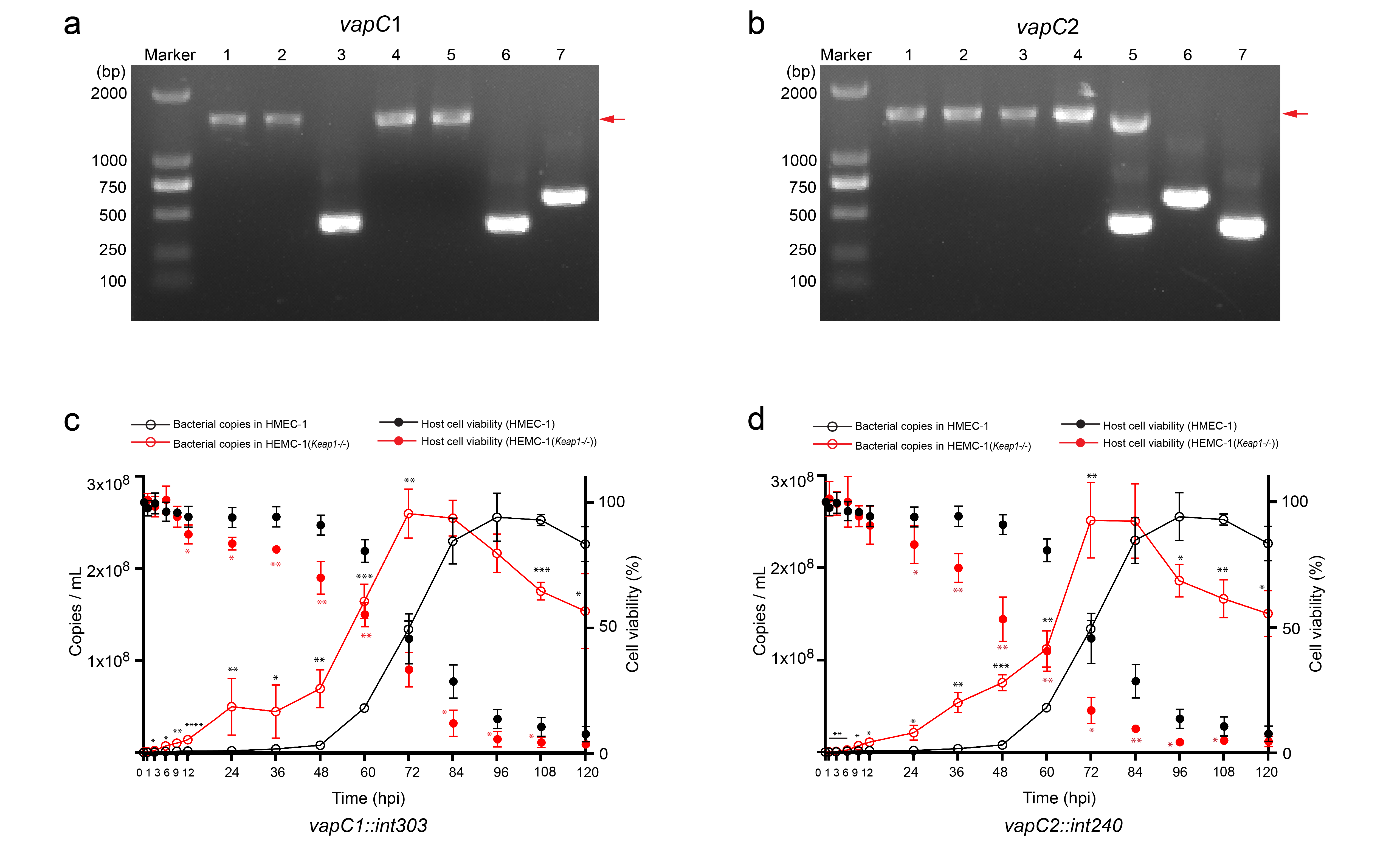

Supplement: S4 Fig — (a,b) PCR amplification of vapC1 and vapC2 genes from representative transformants following plaque selection and expansion. The amplified products showed bands approximately 1500 bp larger than wild-type (arrows), confirming correct targeted insertion. These bands were gel-purified and subjected to direct sequencing. (c, d) Growth kinetics and host cell viability during infection with (c) vapC1::int303, and (d) vapC2::int240 strain in HMEC-1 and HMEC-1 (Keap1-/-) cells. Data represent mean ± SD from three independent experiments. Statistical significance (*p < 0.1, **p < 0.01, ***p < 0.001) was determined by two-tailed unpaired t-test comparing Keap1-/- to wild-type HMEC-1 cells. (TIF) [file ppat.1013380.s004.tif]

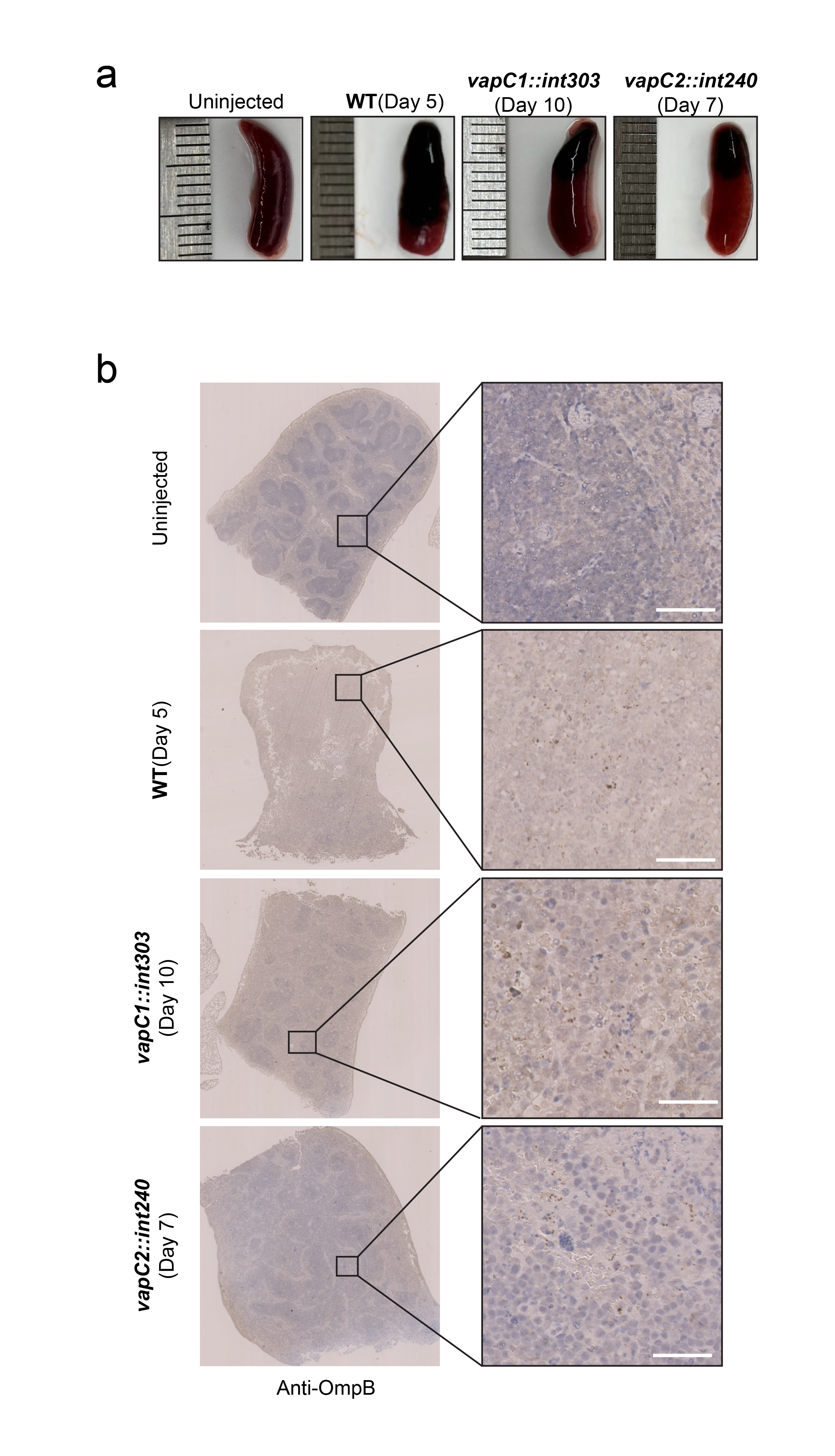

Supplement: S5 Fig — (a) The representative images of spleen tissue from Ifnar1-/- mice that died of infection with WT, vapC1::int303, and vapC2::int240 strain via intravenous route were shown. (b) The representative views of immunohistochemical staining on spleen tissue sections. The anti-OmpB antibody was used to indicate the bacteria. Scale bar, 50 µm. (TIF) [file ppat.1013380.s005.tif]

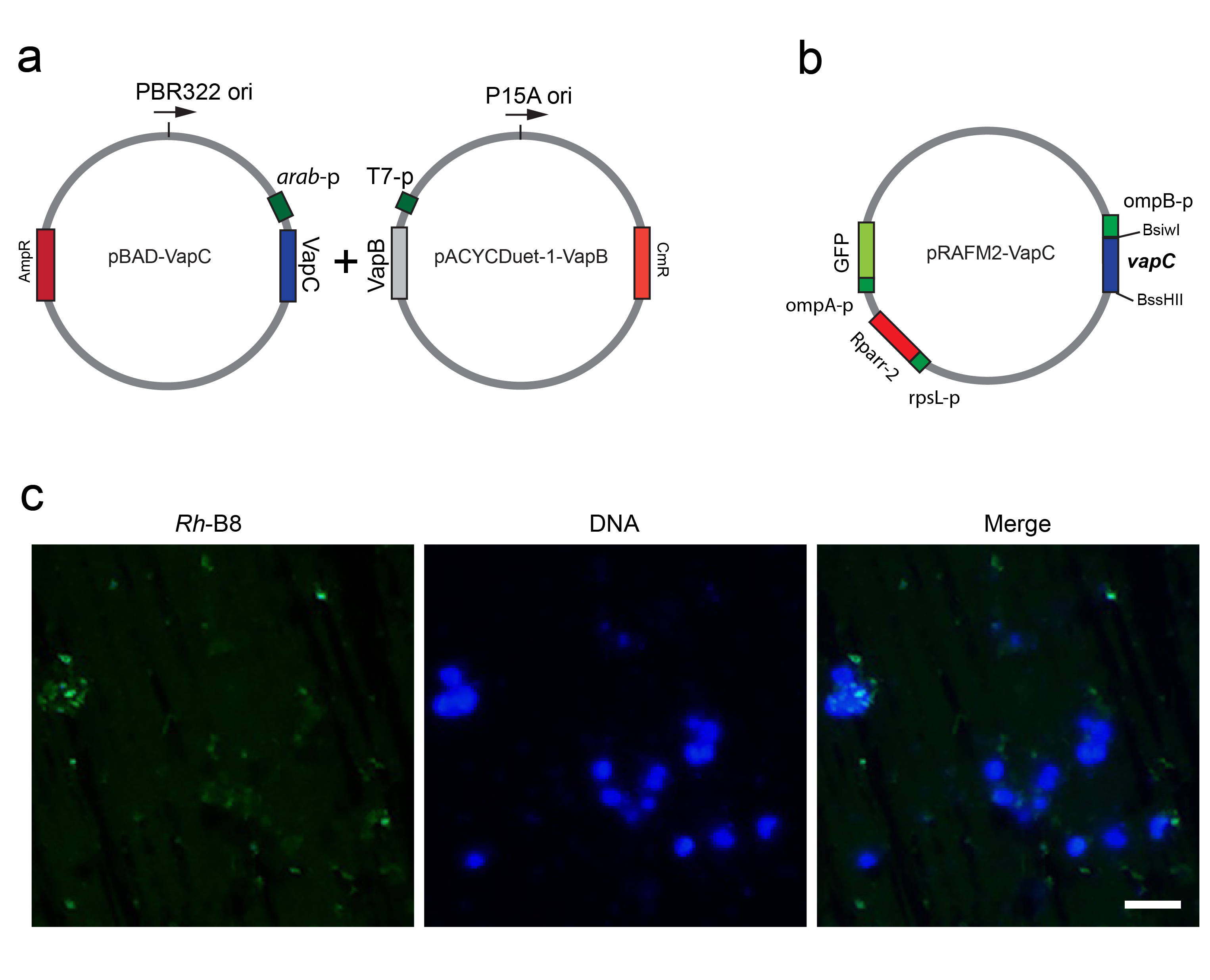

Supplement: S6 Fig — (a) The construction of plasmids for the co-expression of VapB antitoxins and 6 × his tagged VapC toxins in E. coli. (b) The pRAMF2 plasmid for expressing wild-type VapCs and mutants in Rh-B8. The N-terminal FLAG-tagged target proteins are expressed by an ompB promoter, and an ompA promoter expresses GFP. (c) Representative images for transformed Rh-B8 expressing wild-type VapC2 and GFP after plaque selection. Scale bar 10 μm. (TIF) [file ppat.1013380.s006.tif]

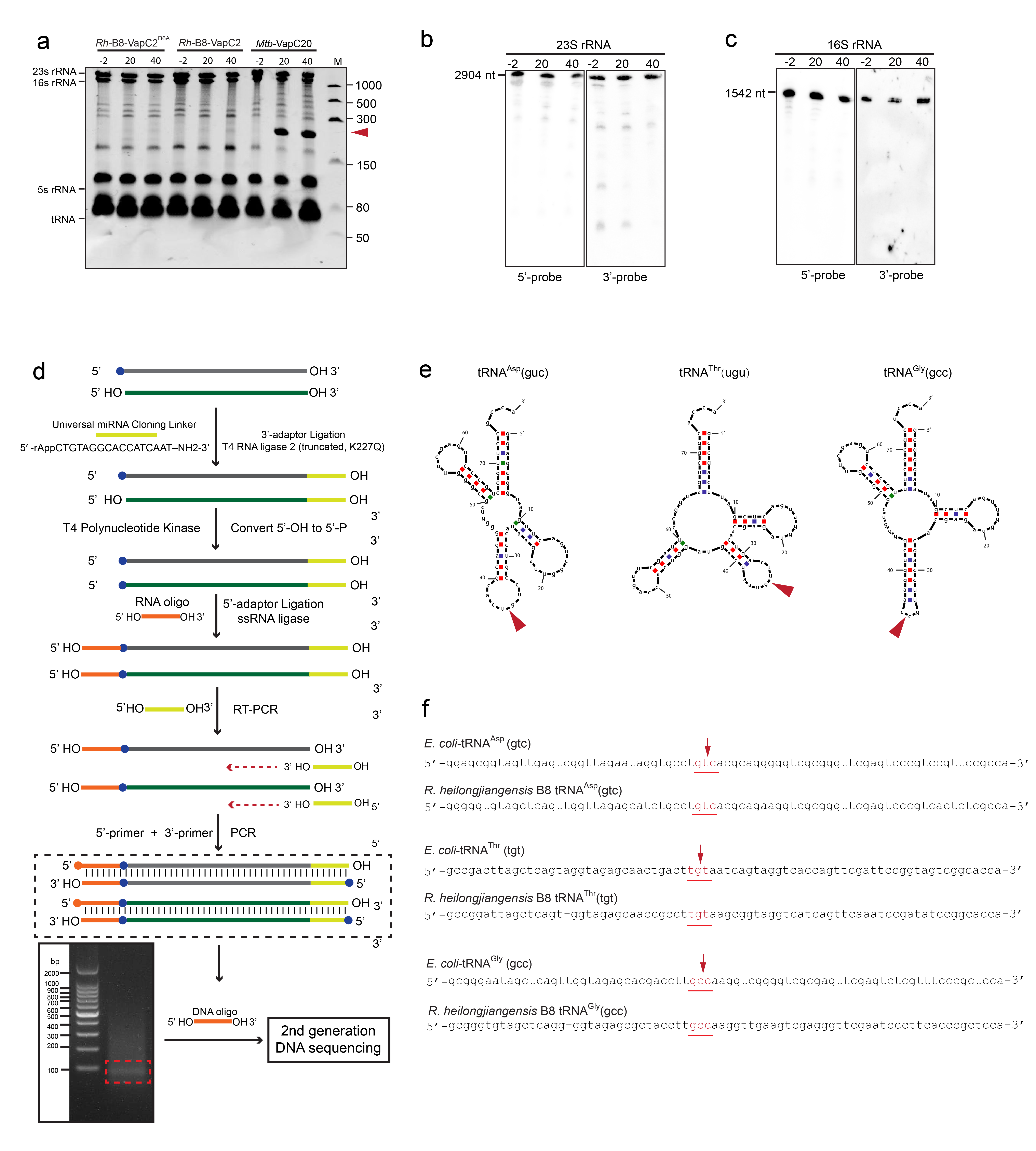

Supplement: S7 Fig — (a) Analysis of total RNA from E. coli after induction of VapC2. E. coli harboring pBAD-VapC2 and pBAD-VapC2D6A were grown in the LB medium, and transcription was induced at time zero by adding L-arabinose (0.2%). VapC20 from M. tuberculosis was used as a control. Cell samples were collected at the indicated time points (min). Total RNA extracted from the samples was separated on a 6% denaturing polyacrylamide gel and visualized by ethidium bromide staining. The VapC20 cleavage products were indicated with an arrow. (b) and (c) Northern blot analysis on the total RNA isolated from E. coli upon induction of VapC2, using probes specific to the 5’ or 3’ sequence of 23S rRNA and 16S rRNA of E. coli. (d) RNA-seq protocol to identify VapC cleavage products as described in Materials and Methods. (e) Secondary structure diagrams of tRNAAsp, tRNAThr, and tRNAGly identified as potential VapC cleavage products. The arrows indicate the site of cleavage based on sequencing results. (f) Sequence alignment of the potential target tRNAs from E. coli and Rh-B8. The anticodon sequences were underlined. (TIF) [file ppat.1013380.s007.tif]

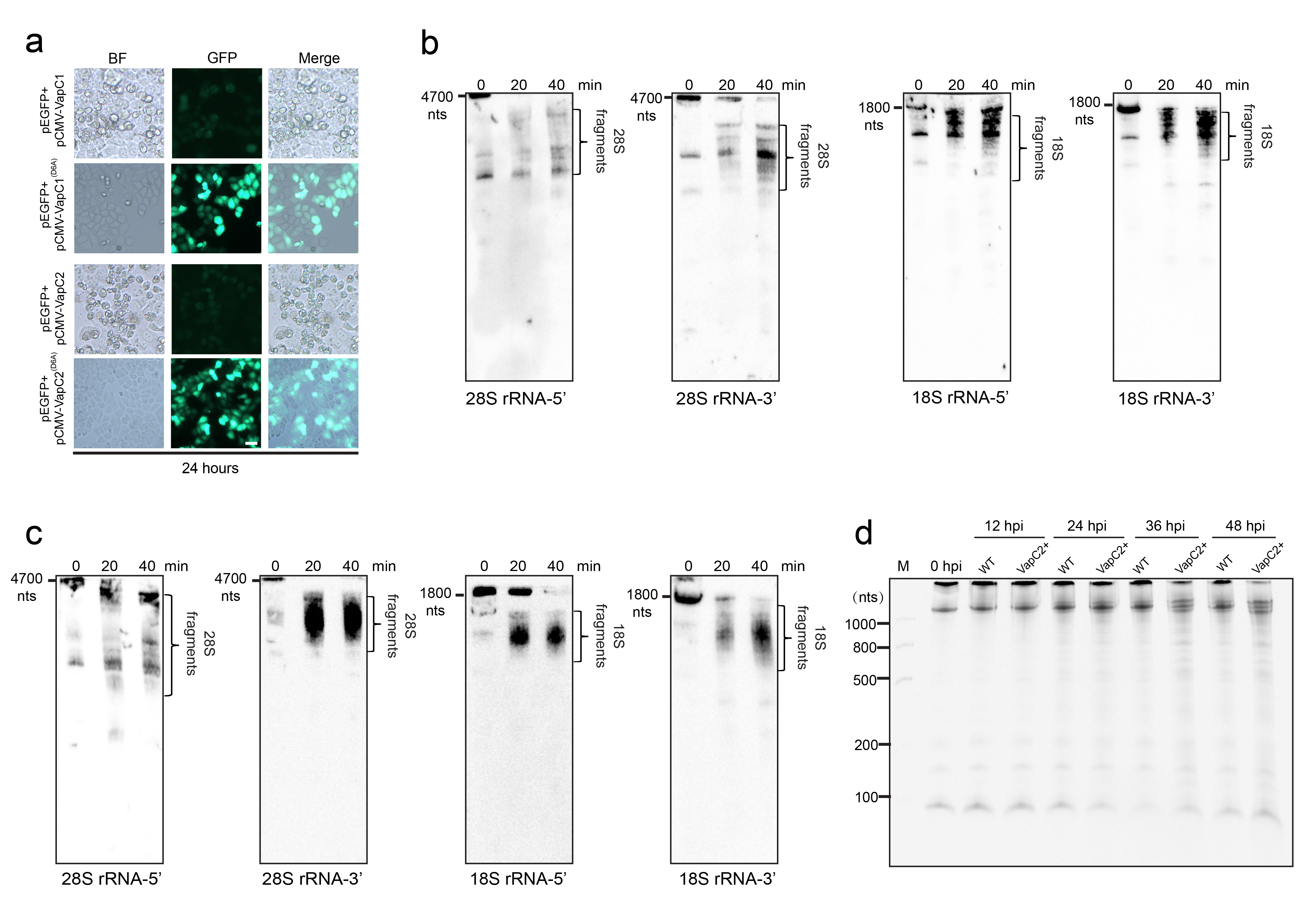

Supplement: S8 Fig — (a) The effect of expression of wild-type VapCs and the inactive mutants in HEK293 cell lines after transformation for 24 hours. The bright field (BF) images show cytopathic effects upon VapCs’ expression. Co-expressed GFP protein was used as a marker for exogenous gene expression. Scale bar, 20 μm. (b) and (c) Total RNA isolated from HMEC-1 was incubated with purified VapCs and then analyzed by Northern blot with probes specific to 5’ or 3’ sequence of 28S rRNA and 18S rRNA from HMEC-1 cells. (d) rRNA degradation in HMEC-1 cells infected with WT or VapC2-overexpressing strains. Total RNA was isolated from infected HMEC-1 cells, separated on a 4.5% denaturing polyacrylamide gel, and stained with ethidium bromide. Enhanced rRNA degradation was observed in cells infected with VapC2-overexpressing strain compared to WT-infected cells. (TIF) [file ppat.1013380.s008.tif]
