# Supplementary material for: VapC toxins promote the pathogenesis of Rickettsia heilongjiangensis by cleaving essential RNAs from both Rickettsia and its host
Source: PLoS Pathog. 2025 Jul 30;21(7):e1013380. doi: 10.1371/journal.ppat.1013380 (PMC12321070; doi:10.1371/journal.ppat.1013380)
Supplement: S1 Table — (DOCX) [file ppat.1013380.s009.docx]

**Table S1 Toxin-antitoxin modules in *Rickettsiae.***

| **Subgroup** | **Species** | **NO. of TA modules (No. of intact TA modules)** | **Annotated intact TA modules** |
| --- | --- | --- | --- |
| **Spotted Fever Group** | *R. conorii (subs. Raoullti)* | 16 (5) | VapB-VapC(2); YefM-YoeB(2); HicA-HicB(1); |
|  | *R. parkeri (str. Atlantic Rainforest)* | 14 (4) | VapB-VapC(2); YefM-YoeB(1); HicA-HicB(1) |
|  | *R. africae (str. ESF-5)* | 14 (4) | VapB-VapC(2); YefM-YoeB(1); HicA-HicB(1) |
|  | *R. rickettsia (str. Iowa)* | 14 (4) | VapB-VapC(2); YefM-YoeB(1); HicA-HicB(1) |
|  | *R. japonica (str. YH)* | 16 (5) | VapB-VapC(2); YefM-YoeB(2); HicA-HicB(1) |
|  | *R. heilongjiangnesis (str. B8)* | 17 (5) | VapB-VapC(2); YefM-YoeB(2); HicA-HicB(1) |
| **Transitional group** | *R. akari (str. Hartford)* | 14 (4) | YefM-ParE(3); ParE-ParD(1) |
|  | *R. australis (str. Cutlack)* | 18 (4) | VapB-VapC(1); YefM-ParE(2); CopG-MazF(1); |
|  | *R. felis (LSU-Lb)* | 27 (10) | VapB-VapC(4); YefM-YoeB(2); HicA-HicB(1); ParE-ParD (1); RelB-YafQ(1); SocA-Txe(1) |
| **Ancestral’ group** | *R. bellii (str. OSU 85-389)* | 25 (10) | VapB-VapC (2); YefM-ParE(2); CopG-MazF (2); YefM-YoeB(2); ParE-ParD (1); BrnA-BrnT(1) |
|  | *R. canadensis (str. CA410)* | 2 (0) | None |
| **Typhus**  **Group** | *R. typhi (str. TH1527, str. TM2540, str. B9991CWPP, str. Wilmington)* | 0 (0) | None |
|  | *R. prowazekii (str. Chernikova, str. Naples-1, str. Breinl, str.Madrid E, str. Katsinyian, str. Dachau)* | 0 (0) | None |
