# Supplementary material for: VapC toxins promote the pathogenesis of Rickettsia heilongjiangensis by cleaving essential RNAs from both Rickettsia and its host
Source: PLoS Pathog. 2025 Jul 30;21(7):e1013380. doi: 10.1371/journal.ppat.1013380 (PMC12321070; doi:10.1371/journal.ppat.1013380)
Supplement: S2 Table — (DOCX) [file ppat.1013380.s010.docx]

**Table S2 Genes and oligonucleotides.**

|  | Name | 5’-3’ sequences |
| --- | --- | --- |
| *Rh*-B8 vapB1 | VapB1^WT^-opt-EC | GGATCCATGAATAAATGGCAACTGCACGAAGCTAAAAATAAACTGTCCAACATTATCGACATTGCAATGCATGGTACTCCGCAATGCATCACCAAACGTGGCGAAGAAGCTGTTGTAATCATCAGCATTAAAGACTACAAACAGCTGACCAAACAGAAGCCGGATTTCAAGGAATATCTGCTGTCCATCCCAAAAACTGATAACCTGGACATTCAGCGTGCAAAAGGTTATGCACGCGACTTCGAACTGTAAAAGCTT |
| *Rh*-B8 vapC1 | VapC1^WT^-opt-EC | GGTACCATGAAATACCTGCTGGACACCAACGTTGTTTCTGAAATCCAGAAAAAAAAATCTAACTCTCAGGTTGCTGCTTGGTTCTCTATCGTTCACTCTTCTCAGCTGTACCTGTCTTGCATCACCATCGGTGAAATCCGTAAAGGTATCTCTAAACTGGCTAAAAAAGACAAAATCGCTTCTCTGAAACTGGAAAAATGGCTGGAACGTATCATCATCGACTACAACGAACGTATCCTGAACATCGGTAAAGAAATCTGCGAAGAATGGGGTGAACTGATGTCTATCGACTCTACCAACGCTATCGACGCTCTGATCGCTGCTCAGGCTAAACAGTCTAACATGATCCTGGTTACCCGTAACGTTAAACACTACAACATGTTCAACATCAAAATCTTCGACCCGTTCAACTAACTCGAG |
| *Rh*-B8 vapB2 | VapB2^WT^-opt-EC | GGATCCGAATTCATGACCCAGATCATCCGTGCTACCGAATTTGTTCGTTCTTTCTCTGACATCATGAACCGTGTTTACTACAAAGGTGAATCTTTCGACGTTCAGAAAGGTAACCACATCGTTGCTCGTATCACCCCGGCTGAAATCAAACCGTCTGTTGCTGTTCGTGACCTGGAAGAAGCGTTCAAAAACGGTCCGCACCTGGACCCGGAAGACGCTGACCAGTTCATGAAAAACCTGGAAGAAATCCGTCGTAACACCAAACAGGACATCAAAAAACTGGTTGAACGTTGGGACTAACTCGAGAAGCTT |
| *Rh*-B8 vapC2 | VapC2^WT^-opt-EC | GGATCCGAATTCATGGGTCTGATCATCGACACCGCTATCATCATCGCTCTGGCTCGTGGTAAAGTTTCTACCAAACAGTGGTCTCACTACGGTCAGACCTACATCTCTCCGATCGTTCTGACCGAACTGCTGATCGGTGTTGACCGTGTTAAAAACGAAAACAAACGTATCAAATGCCTGGCTTTCATCGAATACGTTAAATCTCTGTTCACCATCCTGCCGTTCGGTATCGAAGAAGTTTACACCTACGCTCGTATCACCCACGACCTGTACACCCAGCGTATCACCATCGGTACCCACGACATGCTGATCGCTGCTACCGCTATCACCAAAGGTTTCCCGATCCTGACCCTGAACGTTAAAGACTTCAAACGTATCCAGGGTCTGGAAGTTCTGACCGTTTCTTCTAAAGACTAACTCGAGAAGCTT |
| *Rh*-B8 vapC1 | VapC1^WT^-optfh | GGATCCGAATTCATGAAGTACCTGCTGGACACCAACGTGGTGAGCGAGATCCAGAAGAAGAAGAGCAACAGCCAGGTGGCCGCCTGGTTCAGCATCGTGCACAGCAGCCAGCTGTACCTGAGCTGCATCACCATCGGCGAGATCCGCAAGGGCATCAGCAAGCTGGCCAAGAAGGACAAGATCGCCAGCCTGAAGCTGGAGAAGTGGCTGGAGCGCATCATCATCGACTACAACGAGCGCATCCTGAACATCGGCAAGGAGATCTGCGAGGAGTGGGGCGAGCTGATGAGCATCGACAGCACCAACGCCATCGACGCCCTGATCGCCGCCCAGGCCAAGCAGAGCAACATGATCCTGGTGACCCGCAACGTGAAGCACTACAACATGTTCAACATCAAGATCTTCGACCCCTTCAACTAACTCGAG |
| *Rh*-B8 vapC1 | VapC2^WT^-optfh | GGATCCGAATTCATGGGCCTGATCATCGACACCGCCATCATCATCGCCCTGGCCCGCGGCAAGGTGAGCACCAAGCAGTGGAGCCACTACGGCCAGACCTACATCAGCCCCATCGTGCTGACCGAGCTGCTGATCGGCGTGGACCGCGTGAAGAACGAGAACAAGCGCATCAAGTGCCTGGCCTTCATCGAGTACGTGAAGAGCCTGTTCACCATCCTGCCCTTCGGCATCGAGGAGGTGTACACCTACGCCCGCATCACCCACGACCTGTACACCCAGCGCATCACCATCGGCACCCACGACATGCTGATCGCCGCCACCGCCATCACCAAGGGCTTCCCCATCCTGACCCTGAACGTGAAGGACTTCAAGCGCATCCAGGGCCTGGAGGTGCTGACCGTGAGCAGCAAGGACTAACTCGAG |
| *M.tuberculosis vapC20* | Mtb-VapC20 | GGTACCATGATCTTCGTCGACACGTCCTTCTGGGCTGCACTTGGCAACGCCGGCGACGCTCGGCACGGCACCGCGAAGAGGTTGTGGGCCAGCAAACCGCCCGTGGTGATGACCTCCAACCATGTCCTGGGCGAGACCTGGACGCTGCTCAACCGGCGCTGCGGTCACCGCGCGGCGGTTGCCGCCGCTGCAATCCGCTTGAGCACCGTCGTTCGCGTCGAGCACGTAACAGCCGACCTGGAAGAGCAGGCATGGGAATGGCTTGTACGCCACGACGAGCGAGAGTACTCGTTCGTCGATGCCACGAGCTTCGCGGTGATGCGAAAGAAAGGCATCCAAAATGCGTATGCCTTCGATGGTGACTTCAGCGCGGCCGGATTTGTCGAGGTAAGACCCGAGTAGCTCGAG |
|  | B8-OmpB-frag-RTP | GTTTAATACGTGCTGCTAACCAAGATTATGTAATAACGCGTACTAACAATGCAGAAAACGTAGTTACTAATGATATCGCAAATAGTCCGTTTGGAGGTGCACCGGGTATAGGTCAGAACGTTACAACATTTGTAAATGCAACTAATACTGCAGCATATAATAATCTTCTTTTAGCTAAAAATAGTGCTGATTCTGCTAATTTTGTCGGAACTATCGTTACCGATACAAGTGCGGCCATAACTAATGCACAATTAGATGTAGCTAAAGATATCCAAGCTCAACTTGGTAACAGATTAGGTGCTCTTAGATATTTAGGTACTCCTGAAATGGTTGGATCTGAAGCGGGAGCAATACCGGCTGCGGTTGCTGCAGGTGACGAGGCTGTTGATAATGTAGCTTACGGTATATGGGCAAAACCTTTCTATACTGATGC |
| *Rh*-B8 *vapB1* and truncates | Fw_B8-VapB1-wt | CGGAATTCAATAAATGGCAGTTAC |
|  | Rv_B8-VapB1-wt | CCGCTCGAGTCATAATTCAAAATCTCTAGC |
|  | Rv_N53-B1 | CCGCTCGAGATCAGGTTTTTGTTTAG |
|  | Rv_N40-B1 | CCGCTCGAGACTAATAATTACAACTGC |
|  | Fw_54C-B1 | CGGAATTCTTCAAAGAATATTTATTG |
|  | Fw_41C-B1 | CGGAATTCATAAAAGACTATAAAC |
|  | Fw_41-53-B1 | AATTCATAAAAGACTATAAACAACTTACTAAACAAAAACCTGATC |
|  | Rv_41-53-B1 | TCGAGATCAGGTTTTTGTTTAGTAAGTTGTTTATAGTCTTTTATG |
| *Rh*-B8 *vapB2* and truncates | Fw_B8-VapB2-wt | CGGAATTCACACAAATTATTAGAGC |
|  | Rv_B8-VapB2-wt | CCGCTCGAGTTAATCCCATCTTTC |
|  | Rv_N81-B2 | CCGCTCGAGTTAGCGTCTTATTTC |
|  | Rv_N66-B2 | CCGCTCGAGTTAAGGATCAAGATG |
|  | Rv_N42-B2 | CCGCTCGAGTTAAGGCGTAATCCTTG |
|  | Fw_82C-B2 | AATTCAATACTAAACAAGATATTAAAAAATTGGTTGAAAGATGGGATTAAC |
|  | Rv_82C-B2 | TCGAGTTAATCCCATCTTTCAACCAATTTTTTAATATCTTGTTTAGTATTG |
|  | Fw_66C-B2 | CGGAATTCCCTGAAGATGCTGATC |
|  |  |  |
|  | Fw_42C-B2 | CGGAATTCCCTGCAGAAATCAAG |
|  | Fw_66-81-B2 | AATTCCCTGAAGATGCTGATCAATTCATGAAAAATCTCGAAGAAATAAGACGCTAAC |
|  | Rv_66-81-B2 | TCGAGTTAGCGTCTTATTTCTTCGAGATTTTTCATGAATTGATCAGCATCTTCAGGG |
|  | Fw_42-65-B2 | AATTCCCTGCAGAAATCAAGCCTTCTGTAGCAGTAAGAGACTTAGAGGAAGCTTTTAAAAATGGTCCACATCTTTAAC |
|  | Rv_42-65-B2 | TCGAGTTAAAGATGTGGACCATTTTTAAAAGCTTCCTCTAAGTCTCTTACTGCTACAGAAGGCTTGATTTCTGCAGGC |
| *Rh*-B8 *La* | Fw_La | CGGAATTCATGAATAAAAAATCCCTTCCG |
|  | Rv_La | CCGCTCGAGCTATTTATTTATCGGTGTTC |
| *Rh*-B8 vapC1^WT^ | Fw_VapC1-wt-X | CCGCTCGAGATGAAATACCTGCTGG |
|  | Rv_VapC1-wt-E | CGGAATTCTTAGTTGAACGGGTC |
| *Rh*-B8 vapC2^WT^ | Fw_VapC2-wt-X | CCGCTCGAGATGGGTCTGATCATC |
|  | Rv_VapC2-wt-E | CGGAATTCTTAGTCTTTAGAAGAAAC |
| *Rh*-B8 vapC1^D6A^ | Fw_VapC1-D6A-X | CCGCTCGAGATGAAATACCTGCTGGCAACCAACGTTGTTTCT |
| *Rh*-B8 vapC2^D6A^ | Fw_VapC2-D6A-X | CCGCTCGAGATGGGTCTGATCATCGCAACCGCTATCATCATC |
| Rh_B8 vapC expression in Rickettsia | Fw_VapC1-wt-BW | CCGCGTACGATGAAATACCTGCTGG |
|  | Rv_VapC1-wt-BH | CGGCGCGCTTAGTTGAACGGGTC |
|  | Fw_VapC2-wt-BW | CCGCGTACGATGGGTCTGATCATC |
|  | Rv_VapC2-wt-BH | CGGCGCGCTTAGTCTTTAGAAGAAAC |
|  | Fw_VapC1-D6A-BW | CCGCGTACGATGAAATATTTATTAGCAACTAATGTTGTATCT |
| *Rh*-B8 vapC expression in human cell lines | Rv_VapC1-optfh-X | CCGCTCGAGTTAGTTGAAGGGGTCG |
|  | Rv_VapC2-optfh-X | CCGCTCGAGTTAGTCCTTGCTGCTC |
|  | Fw_VapC1-optfh-D6A-B | CGGGATCCATGAAGTACCTGCTGGCAACCAACGTGGTGAGC |
|  | Fw_VapC2-optfh-D6A-B | CGGGATCCATGGGCCTGATCATCGCAACCGCCATCATCATC |
| *Rh*-B8 vapC1-targetron | 303\|304s-EBS1d | CAGATTGTACAAATGTGGTGATAACAGATAAGTCATCGATGCTAACTTACCTTTCTTTGT |
|  | 303\|304s-EBS2 | TGAACGCAAGTTTCTAATTTCGGTTGCATTCCGATAGAGGAAAGTGTCT |
|  | 303\|304s-IBS | AAAAAAGCTTATAATTATCCTTAAATGCCATCGATGTGCGCCCAGATAGGGTG |
|  | EBS-universal | CGAAATTAGAAACTTGCGTTCAGTAAAC |
| *Rh*-B8 vapC2-targetron | 240\|241s-EBS1d | CAGATTGTACAAATGTGGTGATAACAGATAAGTCTATGCACGTAACTTACCTTTCTTTGT |
|  | 240\|241s-EBS2 | TGAACGCAAGTTTCTAATTTCGGTTGTATATCGATAGAGGAAAGTGTCT |
|  | 240\|241s-IBS | AAAAAAGCTTATAATTATCCTTATATACCTATGCAGTGCGCCCAGATAGGGTG |
| Primers for transformant identification | GFP-F | CTTTTCACTGGAGTTGTC |
|  | GFP-R | CTGCCGTGATGTATACATTG |
|  | arr-F | ATGGTAAAAGATTGGATTCC |
|  | arr-R | TCTTCAATAACATGTAAACCAC |
| Primers used in More-seq | 3’-RNA-adaptor | rAppCTGTAGGCACCATCAAT |
|  | 5'-RNA-adaptor | GUUCAGAGUUCUACAGUCCGACGAUC |
|  | 3‘-RT-Primer-BYT | ATTGATGGTGCCTACAG |
|  | 5’-primer-MS | GTTCAGAGTTCTACAGTCCGAC |
| 5’-digoxin-labeled oligonucleotide probes | B8-tRNA-fMet-1 | DIG-GAGCTACCGAGCTGCTCCACC |
|  | B8-tRNA-fMet-2 | DIG-CGCTCTAACCAGCTGAGCTACC |
|  | B8-tRNA-Asp | DIG-GATGCTCTAACCAACTGAGCTAC |
|  | B8-tRNA-Thr | DIG-GGTTGCTCTACCACTGAGC |
|  | B8-tRNA-Gly | DIG-GTAGCGCTCTACCCCTGAGC |
|  | B8-5S-rRNA | DIG-CGGGATGGGATCGTGTGTTTCACTC |
|  | EC-16S-5 | DIG-GCCAGCGTTCAATCTGAGCCATG |
|  | EC-16S-3 | DIG-CCAACCGCAGGTTCCCCTAC |
|  | EC-23S-5 | DIG-GACTGCCAGGGCATCCACCG |
|  | EC-23S-3 | DIG-GGTTCATTAGTACCGGTTAG |
|  | H-28S-5 | DIG-CTCCGCTGACTAATATGCTTAAATTCAGCG |
|  | H-28S-3 | DIG-GTCGAGGGCTGACTTTCAATAGATCGC |
|  | H-18S-5 | DIG-CTTAATCTTTGAGACAAGCATATGCTACTGG |
|  | H-18S-3 | DIG-GTTCACCTACGGAAACCTTGTTACGAC |
| *ompB*-RTP | Fw-RTP-B8-OmpB | CATCAGTATAGAAAGGTTTTGCCCATA |
|  | Rv-RTP-B8-OmpB | ATCTGAAGCGGGAGCAATACC |
|  | Probe-B8-OmpB | FAM-TACATTATCAACAGCCTCGTCA-BHQ |
| *yefM-yoeB-1*-RTP | Fw-RTP-B8-yefM1 | GCGGAGGATTTAGTGTATTG |
|  | Rv-RTP-B8-yefM1 | CGTGGTTAATTCTACGTGAC |
|  | Fw-RTP-B8-yoeB1 | ACCGTAGAAAACGATCATGTCC |
|  | Rv-RTP-B8-yoeB1 | TTCGATTCGCATTAGCCAGAT |
| *yefM-yoeB-2*-RTP | Fw-RTP-B8-yefM2 | AGCGAAGCAAGAAGTAAGCTTT |
|  | Rv-RTP-B8-yefM2 | GCGCGTCCTTCAATTAGAGAC |
|  | Fw-RTP-B8-yoeB2 | ATTCGTTATACTACACAAGCACA |
|  | Rv-RTP-B8-yoeB2 | ATAACAACCGCTAAAGTCACC |
| *vapBC2*-RTP | Fw-RTP-B8-VapB2 | AGAGCAACAGAATTTGTACGAT |
|  | Rv-RTP-B8-VapB2 | GCAGGCGTAATCCTTGCTAC |
|  | Fw-RTP-B8-VapC2 | CGGTCAGACTTATATTAGCCCTATTG |
|  | Rv-RTP-B8-VapC2 | AAACCTTTTGTAATAGCCGTTGC |
| *hicAB*-RTP | Fw-RTP-B8-HicA | GCAAAAAGGTGGCCATCAGA |
|  | Rv-RTP-B8-HicA | TTGCCATATTCCCAAAAGTGTGA |
|  | Fw-RTP-B8-HicB | GCCATTCGCCTGAAAAAGCA |
|  | Rv-RTP-B8-HicB | TCCTAGACGGAGCTAAGGGA |
| *vapBC1*-RTP | Fw-RTP-B8-VapB1 | TGGCAGTTACACGAAGCAAAG |
|  | Rv-RTP-B8-VapB1 | CAACTGCTTCTTCACCCCTT |
|  | Fw-RTP-B8-VapC1 | ATTCTCAAGTAGCAGCATGGTT |
|  | Rv-RTP-B8-VapC1 | ACTCCTCACATATTTCTTTGCCAA |
| *clpX-*RTP | Fw-RTP-B8-ClpX | CGGGCAATTTTATCAACCTCGT |
|  | Rv-RTP-B8-ClpX | ACCTTTTACTATGGCGGATGCT |
| *lon*-RTP | Fw-RTP-B8-Lon | CCTTGGATCAATGCCCGGTA |
|  | Rv-RTP-B8-Lon | ACCTCAAGCAAAGCGGAT |
| *Keap*1 knock out | Keap-KO-1-up | CACCGccgtgtaggcgaattcaatg |
|  | Keap-KO-1-down | AAACcattgaattcgcctacacggC |
|  | Keap-KO-2-up | CACCGagatgtactcccgggcacgc |
|  | Keap-KO-2-down | AAACgcgtgcccgggagtacatctC |
