# Supplementary material for: VapC toxins promote the pathogenesis of Rickettsia heilongjiangensis by cleaving essential RNAs from both Rickettsia and its host
Source: PLoS Pathog. 2025 Jul 30;21(7):e1013380. doi: 10.1371/journal.ppat.1013380 (PMC12321070; doi:10.1371/journal.ppat.1013380)
Supplement: Attachment — Submitted filename: Response_to_Reviewers.docx [file ppat.1013380.s012.docx]

Full title: VapC toxins promote the pathogenesis of *Rickettsia heilongjiangensis* by cleaving essential RNAs from both *Rickettsia* and its host

Short title: VapBC TA modules and the pathogenesis of SFG *Rickettsia*

**Point-by-Point Response to Reviewers’ Comments**

We sincerely appreciate the comments and suggestions from the editor and reviewers. Below we provide point-by-point responses to each concern, along with corresponding revisions in the manuscript.

**Part I - Summary**
**Reviewer #1**: In this manuscript, Liu et al. characterize a newly discovered toxin-antitoxin system in Rickettsia that enables the bacteria to withstand host reactive oxygen species. The study elegantly demonstrates that these systems are expressed during the early stages of Rickettsia infection and that VapBC1 is secreted and plays a crucial role in pathogenesis and resistance to host ROS. The authors provide compelling evidence that VapBC1 exerts its function by cleaving the anti-codon loop of tRNAfMet, whereas VapBC2 nonspecifically degrades host rRNA. Overall, this is a well-executed and rigorously controlled study. My comments are minor and largely cosmetic.

Authors: We sincerely appreciate the reviewer's positive comments of our research.

**Reviewer #2**: Toxin-antitoxin (TA) modules enable bacteria to persist under stressful environments. In this manuscript, authors identified and characterized 2 TA system: vapBC1 and vapBC2 from an intracellular pathogen R. rickettsii. The data showed that vapBC1 is crucial for Rickettsia to withstand accumulated host reactive oxidative species (ROS), via induction of bacterial dormancy through cleavage on the anti-codon loop of tRNAfMet, thereby facilitating intracellular survival and infection in a mouse model. Another vapBC module (vapBC2) was found to be activated and toxin exposed to host cytoplasm, contributing to Rickettsia's virulence and adaptability in its human host by non-specifically degrading host rRNAs rather than regulating rickettsial growth.

Overall, the experiments are well designed, data is solid, the discovery is novel and significant.

Authors: We sincerely appreciate the reviewer's positive comments of our research.

Minor comments:

1) In the result section "*Rh*-B8 VapBCs have distinctive intermolecular...". These data were generated from predicted models and interactions, the conclusion should be interpreted with caution (i.e., softening the conclusions)
Authors: Thank you for your suggestion. To present our conclusions more conservatively, we have thoroughly revised the results section titled '*Rh*-B8 VapBCs have distinctive intramolecular interaction patterns' in the updated manuscript (Lines 157-178).

2) What is homology level between vapB1 and vapB2?
Authors: To our knowledge, VapB antitoxins from different bacterial VapBC toxin-antitoxin modules share no detectable sequence homology. In line with this observation, our sequence alignment analysis showed no significant similarity between the two *Rh*-B8-encoded VapB proteins. Likewise, although VapC toxins can be identified by conserved active-site residues, they exhibit no appreciable sequence similarity outside these residues.

3) in some result sections, VapC1 or VapC2 should be clearly indicated, rather than VapC toxins.

Authors: The toxin component referring to VapC1 or VapC2 is now explicitly indicated in the revised paper, and all references to the two VapC toxins have been revised to "both VapC toxins" (including both VapC1 and VapC2).

**Reviewer #3**: The manuscript by Liu et al. describes the identification and initial characterization of two toxin-anti-toxin modules, VapBC1 and VapBC2 in the spotted fever group Rickettsiae. Both modules were induced during infection with a matched induction of expression of the rickettsial Lon protease. The authors identified Lon as the protease responsible for cleaving the antitoxin modules VapB1 and VapB2, thus respectively freeing VapC1 and VapC2. The authors also provided evidence for the specific interactions of VapB1 with VapC1 and of VapB2 with VapC2. Both protease and VapBC modules were transcriptionally induced in response to exposure to H_2_O_2_. Additional characterization revealed the secretion of VapC2 to the host cell cytosol, where it cleaves a ribosomal RNA component, while VapC1 was retained in the bacterial where it apparently cleaves fMet-tRNA. The former was speculated to be involved in host cell cytotoxicity, while the latter was hypothesized to arrest bacterial growth in times of oxidative stress. Significance of the VapBC modules were characterized using two assays – plaque formation and quantification of plaque size and in a mouse model of infection where body weight, body temperature, and death were monitored.

Overall, the authors provided evidence that demonstrated the potential pathogenic significance of both VapBC1 and VapBC2, along with mechanisms of how the toxin components are liberated, i.e. through the proteolytic action of Lon on the respective anti-toxin components. With regards to the mechanism of VapC1 cleavage of fMet-tRNA, evidence for this mode of action was primarily obtained from tRNA profiling of E. coli ectopically expressing VapC1, which was followed by in vitro cleavage experiments focused on rickettsial fMet-tRNA, and the identification of cleavage products by northern blot using an fMet-tRNA-specific probe. This is crucial data, which I commend the authors for providing. The authors further strengthened the tRNA cleavage function of VapC1 by demonstrating the lack of growth effects of a predicted catalytically dead VapC1 in both E. coli and Rickettsia.

What the manuscript failed to establish convincingly is the possible cause-and-effect relationship between rickettsial growth inhibition mediated by VapC1-dependent fMet-tRNA degradation and reduced ROS burden. The authors attributed this reduction to host response, and not considering a reduction due to growth arrest of rickettsiae.

Authors: Thank you for raising this critical question regarding whether rickettsial growth arrest—triggered by tRNA^fMet^ degradation—directly contributes to the observed reactive oxygen species (ROS) fluctuations in HMEC-1 cells. We have carefully addressed these points in the revised manuscript, as detailed below (**Part II**) in our point-by-point responses and corresponding textual modifications.

**Part II – Major Issues: Key Experiments Required for Acceptance**
**Reviewer #1**: None noted

**Reviewer #2**: Overall, the experiments are well designed, data is solid, the discovery is novel and significant.

Authors: We sincerely appreciate the reviewer's positive comments of our research.

**Reviewer #3**: Issues mentioned below are related to the need to clarify any potential link between bacterial growth arrest and reduced ROS burden. The authors attributed this to the induction of host response. The reduction could also be related to growth-arrested bacteria.
Authors: This portion of our study focuses on host environmental challenges encountered by *Rickettsia* as an obligate intracellular pathogen, specifically examining key stressors that activate *vapBC* toxin-antitoxin (TA) modules in *Rh*-B8. We identified reactive oxygen species (ROS) burden - a crucial component of endothelial cell-mediated anti-infection immunity - as a potent inducer of both *vapBC* operons.

Time-course analysis revealed an immediate post-infection surge followed by gradual resolution to baseline levels by 24 hours post-infection (hpi), coinciding with bacterial growth recovery. The sustained absence of ROS elevation during subsequent rickettsial proliferation supports our hypothesis that ROS levels and bacterial growth may represent independent processes. Notably, coordinated increases in glutathione (GSH) concentration and antioxidant enzyme expression prompt us to conclude that ROS clearance is primarily mediated through host defense mechanisms.

We sincerely appreciate the reviewer's observation regarding the potential role of the VapBC modules in modulating ROS dynamics. This valuable perspective, which was not fully addressed in our initial study, certainly merits further investigation to elucidate the complex interplay between bacterial TA systems and host oxidative defenses.

The experiments conducted to generate data in Fig. 3E, where the authors measured ROS burden in mock- and infected cells over time should be confirmed with their various mutant strains to clarify potential direct role of the pathogen in the reduction in ROS levels. ROS levels could be monitored early in infection (0-12 h) with respect to infection with wild type and various VapBC mutant strains. Currently, it’s not clear what causes this reduction in ROS burden. Is it due to reduced pathogen replication via VapC1 action, or is it related to the general inhibition of host translation caused by VapC2? Is there a correlation between the rates of rRNA cleavage and ROS burden?

Authors: Thank you for your suggestion. We monitored host ROS levels during VapC mutant strains infection. Both wild-type (WT) and Δ*vapC1* infections exhibited similar ROS profiles - an initial elevation followed by rapid decline (updated Figure 7i) - along with comparable temporal upregulation of antioxidant enzymes (updated Figure 7j). These findings suggested that host ROS generation and clearance occur independently of VapC1's growth-suppressive function. Notably, Δ*vapC1* mutant strain showed attenuated growth in HMEC-1 cells, potentially resulting from uncontrolled proliferation and consequent increased susceptibility to ROS. Its cellular ROS profiles remained indistinguishable from the wild-type strain.

We acknowledge the reviewer's insightful observation that VapC1-mediated dormancy in *Rh*-B8 may lead to misinterpretation of transient growth arrest as complete infection resolution, thereby potentially masking actual host ROS production. In response to this valuable suggestion, we have revised our discussion (lines 490-504) to incorporate these considerations.

Interestingly, Δ*vapC2*-infected cells displayed accelerated ROS clearance compared to both WT and Δ*vapC1* strains (updated Figure 7i). Given VapC2's established role in host translational inhibition, we hypothesized it might impair host antioxidant defenses. Supporting this, infection with VapC2-overexpressing strains caused: prolonged ROS accumulation (updated Figure 7i), and enhanced host rRNA degradation (updated Figure S8d). These results indicate that VapC2's broad translational suppression likely compromises the host's antioxidant capacity. All new data have been systematically incorporated into the revised manuscript (lines 431-449 in text file, and updated Figure 7 and Figure S8).

These new findings are further evidenced by our analysis showing that VapC2 exhibits stronger host-interaction capacity than VapC1, including greater mammalian cell cytotoxicity (Figure 7b-d) and more efficient rRNA degradation (Figure 7e-f). Notably, unlike the VapC2-overexpressing strain, wild-type infections likely maintain tight regulation of VapC2 expression and secretion, allowing for precise host manipulation. Therefore, we’ve also revised our discussion to reflect these findings and have been careful to qualify our interpretations accordingly (lines 490–504).

Another crucial aspect of the model proposed that was not addressed was how the pathogen recovers, i.e. how normal growth rate is restored or how it exits from growth arrest/inhibition after oxidative stress attenuation by the VapBC system; and when recovery happens during infection. Replenishing of the fMet-tRNA pool should also be monitored in experiments that address recovery.
Authors: Thank you for your comment. We demonstrated that *Rh*-B8's growth recovery could result from two coordinated mechanisms: (1) Neutralization of VapC1 toxicity through binding of its cognate antitoxin VapB1, which occurs once Lon protease-mediated VapB1 degradation is reduced (VapB1 is inherently overexpressed relative to VapC1 due to its promoter-proximal position in the operon); (2) When oxidative stress is resolved, transcriptional silencing of the *vapBC* operon halts VapC1 production, likely through a mechanism involving VapBC complex binding to the operon's promoter.

In response to the reviewer's suggestion, we analyzed the tRNA^fMet^ pool dynamics during infection, given our discovery that initiator tRNA^fMet^ is the primary target of VapC1. Time-course experiments revealed complete restoration of tRNA^fMet^ levels following initial depletion, with this recovery profile precisely mirroring *Rh*-B8's growth kinetics. These data establish that the early infection growth delay directly results from VapC1-mediated cleavage of initiator tRNA^fMet^. These results have been incorporated into the revised manuscript (Lines 382-387 in revised manuscript and updated Figure 6h-j).

To further implicate an oxidative stress-related function for VapC1, mutant cells unable to generate reactive oxygen species in response to rickettsia infection could be used to evaluate the growth dynamics of wild type and mutant rickettsial strains.
Authors: We sincerely appreciate reviewer's suggestion. Our follow-up experiments confirmed significantly reduced transcription of *vapB1* and *vapB2* during early *Rh*-B8 infection in *Keap1*-knockout HMEC-1 cells, which exhibit impaired ROS production due to constitutive antioxidant enzyme overexpression (see updated Figure 3e and f, and Lines 199-205 in revised manuscript).

We further characterized the growth kinetics of wild-type and mutant *Rh*-B8 strains in *Keap1*-knockout HMEC-1 cells. All strains exhibited enhanced replication rates during infection, indicating that VapC-mediated growth regulation of *Rh*-B8 is not fully activated under oxidative stress-deficient conditions (see updated Figure 4e, Figure S4 c and d, and Lines 275-285 in revised manuscript).

With regards to the observed cytotoxicity of VapC2 on host cells, and subsequent data that demonstrated a role for VapC1 in the degradation of host rRNAs, it seems contradictory when viewed in the context of the proposed host response to minimize oxidative damage. The preservation of this response was based on the observed basal level of ROS burden at later time points in infection, and the expression of several markers of host proteins that deal with oxidative stress. Could this be due to growth arrest of rickettsia in the presence of oxidative stress? In other words, would a VapC1 mutant strain, which is predicted to be incapable of growth arrest sustain relatively high ROS burden levels?

Authors: VapC2's rRNA-degrading activity may exert broad physiological effects. Consequently, its secretion into host cytoplasm must be tightly regulated to prevent excessive host translational arrest while partially disrupting antibacterial responses. Given these considerations, we keep caution in interpreting VapC2's cytotoxicity data as stated above (also see Discussion, lines 490–504).

Yes, we have stated this point that VapC1-induced bacterial dormancy may give a false impression of infection resolution while potentially promote to retard host ROS production. Notably, host ROS levels remain suppressed even during subsequent rickettsial growth recovery. The observed activation of host antioxidant responses suggests this sustained ROS suppression is mediated by host antioxidant mechanisms. We believe that host ROS moderation represents an evolutionarily optimized strategy benefiting *Rickettsia*, as excessive host cell damage would ultimately impair rickettsial proliferation.

Our experiments demonstrated that the Δ*vapC1* mutant strain grow equivalent well as wild-type in *Keap1*-knockout HMEC-1 cells (Fig. 4e, Fig. S4 c and d). However, Δ*vapC1* mutant strain exhibited significantly reduced survival under high H_2_O_2_ exposure (Figure 4f). This impaired oxidative stress resistance suggests that growth arrest-deficient strains cannot effectively manage ROS accumulation, as uncontrolled replication increases bacterial vulnerability to oxidative damage.

**Part III – Minor Issues: Editorial and Data Presentation Modifications**

**Reviewer #1**:

Figure 2A: The labeling needs improvement. What does "S# →" represent (β-sheet)? What does the "L" with the dotted bracket indicate?

Authors: Thank you for identifying this need for clarification. The secondary structure labels in Figure 2 represent: S (β-sheet), H (α-helix), and L (loop regions). We have revised the figure legend to explicitly state these designations.

Additionally, in panels D and E, the VapC1 bands are difficult to discern. Would it be possible to add a quantification graph?
Authors: Thank you for your suggestion. The quantification of VapC1 and VapC2 bands shown in Figure 2d and 2e has been supplemented with additional data in Figure S2e and S2f.

Figure S3: The gel contains multiple wells labeled 1–7, but their meaning is unclear. These labels should be explicitly defined either in the figure or the legend.
Authors: The figure legend has been updated to clearly indicate the PCR amplification results of *vapC1* and *vapC2* genes from representative transformants following plaque selection and expansion.

Figure 4G and H: These panels are very busy. Is there a more effective way to present this data for improved clarity?

Authors: For full data transparency, all data points were included in these panels. We have optimized visualization by reducing the size of individual data point symbols to improve distinction between overlapping curves while maintaining complete dataset representation.

**Reviewer #2**: see above

**Reviewer #3**: The manuscript would benefit from a more thorough review of grammar. There were several instances in the article where "the" should be omitted. Some examples are line 340 - "may promote the host cell damage...", line 412 - "with the prior analysis...",
Authors: Thank you for pointing out the linguistic concerns. We have thoroughly edited the manuscript to correct all grammatical errors and enhance overall text clarity.

Line 231, "extend" should be "extent".
Authors: Thanks, this has been corrected in the revised manuscript.

Also, "promotor" should be corrected to "promoter".

Authors: Thanks, this has been corrected in the revised manuscript.
